# The MHC Gene Region of Murine Hosts Influences the Differential Tissue Tropism of Infecting *Trypanosoma cruzi* Strains

**DOI:** 10.1371/journal.pone.0005113

**Published:** 2009-04-01

**Authors:** Jorge M. Freitas, Luciana O. Andrade, Simone F. Pires, Ricardo Lima, Egler Chiari, Ricardo R. Santos, Milena Soares, Carlos R. Machado, Gloria R. Franco, Sergio D. J. Pena, Andrea M. Macedo

**Affiliations:** 1 Departamento de Bioquímica e Imunologia, Universidade Federal de Minas Gerais, Belo Horizonte, Minas Gerais, Brazil; 2 Departamento de Morfologia, Universidade Federal de Minas Gerais, Belo Horizonte, Minas Gerais, Brazil; 3 Departamento de Parasitologia , Universidade Federal de Minas Gerais, Belo Horizonte, Minas Gerais, Brazil; 4 Centro de pesquisas Gonçalo Moniz - CPqGM, Salvador Bahia, Brazil; Technical University Munich, Germany

## Abstract

We have previously demonstrated that both parasite genetic variability and host genetic background were important in determining the differential tissue distribution of the Col1.7G2 and JG *T. cruzi* monoclonal strains after artificial infections in mice. We observed that the JG strain was most prevalent in hearts of mouse lineages with the MHC haplotype *H-2* ^d^ (BALB/c and DBA2), while Col1.7G2 was predominant in hearts from C57BL/6 mice, which have the *H-2* ^b^ haplotype. To assess whether the MHC gene region indeed influenced tissue tropism of *T. cruzi*, we used the same two parasite strains to infect C57BL/6 (*H-2* ^b^) and C57BLKS/J (*H-2* ^d^) mice; the latter strain results from the introgression of DBA2 MHC region into the C57BL/6 background. We also performed *ex vivo* infections of cardiac explants from four congenic mice lineages with the *H-2* ^b^ and *H-2* ^d^ haplotypes arranged in two different genetic backgrounds: C57BLKS/J (*H-2* ^d^) versus C57BL/6 (*H-2* ^b^) and BALB/c (*H-2* ^d^) versus BALB/B10-*H2^b^* (*H-2* ^b^). In agreement with our former observations, Col1.7G2 was predominant in hearts from C57BL/6 mice (*H-2* ^b^), but we observed a clear predominance of the JG strain in hearts from C57BLKS/J animals (*H-2* ^d^). In the *ex vivo* experiments Col1.7G2 also prevailed in explants from *H-2* ^b^ animals while no predominance of any of the strains was observed in *H-2* ^d^ mice explants, regardless of the genetic background. These observations clearly demonstrate that the MHC region influences the differential tissue distribution pattern of infecting *T. cruzi* strains, which by its turn may be in a human infection the determinant for the clinical forms of the Chagas disease.

## Introduction

Chagas disease, caused by the protozoan parasite *Trypanosoma cruzi*, has a variable clinical course, ranging from asymptomatic to severe chronic cardiac and/or gastrointestinal disease. The mechanisms involved in this wide range of clinical manifestations of Chagas disease are still not completely understood, but certainly both parasite and host-associated aspects are important [1]. Using the sensitive DNA profiling technique, LSSP-PCR (Low-Stringency single Specific Primer PCR), we previously showedthat hearts of BALB/c mice doubly infected with JG and Col1.7G2 *T. cruzi* populations were mainly colonized by JG strain, while Col1.7G2 was preponderant in the rectum and all other analyzed tissues [2]. This provided strong and direct evidence that parasite genetic variability was involved in the differential tissue tropism and pathology of the infection.

The influence of host genetic factors in this process was revealed subsequently using different mouse strains, by the demonstration that the patterns of parasite tissue distribution were similar for BALB/c and DBA-2 mice, but different for C57BL/6 mice [3]. Since BALB/c and DBA-2 lineages share the MHC haplotype (*H-2* ^d^) and the C57BL/6 lineage has a different haplotype (*H-2* ^b^), we hypothesized that the host MHC gene region might influence the differential tissue distribution of *T. cruzi* strains in these mice. The murine MHC gene region spans approximately 4 Mb of chromosome 17 (23.0 cM, cytoband B–C) and contains 3 major classes of highly polymorphic gene sets: class I (*H-2*-K, *H-2*-D, Q, *H-2*-T18 genes), class II (*H-2*-I genes), and class III (*H-2*-S genes) [4]. These genes are involved in many immunological processes, including graft rejection, immune response, antigen presentation and complement component (http://www.informatics.jax.org).

We have tested the influence of host MHC gene region on differential tissue distribution of *T. cruzi* strains by studying four congenic mice lineages with two different *H-2* haplotypes arranged in two different genetic backgrounds: C57BLKS/J (*H-2* ^d^) versus C57BL/6 (*H-2* ^b^); BALB/c (*H-2* ^d^) versus BALB/B10-*H2^b^* (*H-2* ^b^). Our data showed irrevocably both *in vivo* and *ex vivo* that predominance of one or the other strain of *T. cruzi* (JG or Col1.7G2) in mice heart tissue was dependent on the MHC gene region background, where *H-2* ^b^ haplotype selected for Col1.7G2 clone while *H-2* ^d^ haplotype selected for JG monoclonal population. These observations strongly suggest there is a significant role of host MHC and/or associated genes in the differential rates of growth and tissue distribution pattern of *T. cruzi* strains.

## Materials and Methods

All procedures for animal manipulation and experiments are in accordance with the COBEA, the Brazilian institution that regulates animal experimentation.

### Congenic mice

Male mice (5–6 weeks old) were used in these experiments. Two strains, C57BLKS/J (*H-2^d^*) - Stock Number: 000662 - resulting from the introgression of the DBA2 MHC into the C57BL/6 background and BALB/B10-*H2^b^* – also named C.B10 *H-2* Stock Number: 001952 - in which the BALB/cLilMcdJ MHC region was introgressed into the C57BL/10J MHC gene region, were obtained directly from Jackson Laboratories. C57BL/6 (*H-2*b) mice were obtained from “Centro de Bioterismo” of ICB / UFMG and BALB/c (*H-2* ^d^) were obtained from CPqGM, FIOCRUZ/BA.

### Parasites

For artificial infections we used two different populations of *T. cruzi*: Col1.7G2 (*T. cruzi* I - Zymodeme 1, rDNA group 2, miniexon group 2, mitochondrial haplotype A) and JG (*T. cruzi* II - Zymodeme 2, rDNA group 1, miniexon group 1, mitochondrial haplotype C) originally isolated from patients with distinct forms of Chagas' disease. The JG strain, isolated from the blood of a patient with megaesophagus, was previously typed at eight different microsatellite loci[5] and did not show more than two alleles in any of them, indicating that it is monoclonal (data not shown). Col1.7G2 was cloned from the Colombian strain, which was originally cultured from the blood of a chronic cardiac patient [6]. Infective trypomastigote forms were obtained from blood of infected Swiss mice and diluted to 50 parasites/100 µl of sterile PBS for infection of mice. For infection of heart explants, *T. cruzi* infective trypomastigotes were prepared from the supernatant of LLCMK2 cell cultures infected with each parasite lineage.

### Experimental infections in mice

For infections in mice we used the same protocol described by Andrade [2]. Briefly, mice were intraperitoneally inoculated with a mixture of both parasites (50+50). All infections were done in duplicates at different days.

Six months after infection, corresponding to the chronic phase, animals were killed and samples from the heart and rectum were collected. Age-matched animals were used as controls. Two fragments taken from each organ were washed exhaustively in isotonic saline and stored in ethanol at −20°C. Tissue samples were subjected to alkaline lysis[2] and used directly in the PCR after 10-fold dilution in double-distilled water.

### Cardiac murine explants

Hearts from the four mouse lineages were aseptically removed and sliced at 0.5 mm width in a Tissue Chopper (McIlwain MTC/2 – The Mickle Laboratory engineering Co. LTD.). Two or three slices totaling approximately an area of 10 mm^2^ were exhaustively washed with PBS buffer and deposited over a thin layer of 2% bovine gelatin in DMEM media supplemented with 10% FBS and 50 µg/ml gentamycin in individual wells of a 24-well culture plate, and covered with 2 ml of the same media without gelatin. After 2 h of incubation at 37°C in a 5% CO_2_ chamber, 5×10^5^ trypomastigotes of the JG strain and/or Col1.7G2 clone were added. All wells were washed 24 h later with sterile PBS and fresh media was added to eliminate non-internalized parasites. For PCR analysis, tissue slices were rinsed and collected at 24, 96 and 120 h, submitted to the alkaline lysis protocol[2] and used directly in the PCR after 10-fold dilution in double-distilled water.

### Detection and characterization of parasites by LSSP-PCR of infected tissues

Detection of parasites from each tissue or explant sample was performed by specific PCR amplification of a fragment (about 330 bp) corresponding to the four-variable region of the *T. cruzi* kinetoplast DNA minicircle, as described previously [2]. The PCR products were visualized in a 6% polyacrylamide gel electrophoresis and silver stained as previously described [7]. Characterization of the parasites from the positive tissues by LSSP-PCR was performed as described earlier [8]. Briefly, kDNA amplicons were subjected to electrophoresis in 1.5% agarose gel (1.0% agarose, 0.5% agarose low melting point), punctured from the gel, diluted 10 times in double distilled water, and submitted to a second step of low stringency amplification, using a single primer (S35G: 5′-ATGTACGGGGAGATGCATGA-3′). The LSSP-PCR products were also visualized after a 6% polyacrylamide gel electrophoresis and silver staining.

### Semi-quantitative data from LSSP profiles

For a semi-quantitative analysis, the proportions of the *T. cruzi* strains in each tissue of the doubly infected animals or cardiac explant samples were estimated in a fluorescent automated DNA Sequencer (ALF, Pharmacia Biotech) as described before [2]. For this, the LSSP-PCR was carried out using a fluorescein-labeled S35G primer. The PCR products were then subjected to a 6% polyacrylamide gel electrophoresis under denaturing conditions (8 M urea) and the data obtained were analyzed using the AlleleLinks software (Pharmacia Biotech). The area under specific peaks of each population was calculated and used to evaluate approximately the relative proportions of the JG strain and Col1.7G2 clone by reference to a standard curve. It was demonstrated that LSSP-PCR profiles of equal mixtures of the two strains were the sum of the individual profiles where the specific peak areas were related to the proportions of each population (25∶75; 50∶50; and 75∶25) [3].

Two-sample Student t test was applied to validate differences between values obtained from murine cardiac explants.

### Detection and characterization of parasites in infected tissues by real-time PCR

The characterization of the parasites in infected mice explants was also done by real-time PCR of the D7 region of the rRNA 24S gene. For that, five nanograms of parasite DNA or 5µl of the product of the alkaline lyses obtained from culture explants were used as template in a two-round PCR assay, as described previously [9]. Briefly, samples were first submitted to a PCR using two primers: D75 = 5′– CAGATCTTGGTTGGCGTAG–3′ and D72 = 5′– TTTTCAGAATGGCCGAACAGT–3′). Two microliters of these PCR products were used as template in the second PCR round performed in a real time PCR apparatus (ABI7900 - Applied Biosystems), using the primers: D71 (5′-AAGGTGCGTCGACAGTGTGG-3′) and D72. Since Col1.7G2 and JG D7 regions presented amplicons with TM of 81.5 and 78.2°C, respectively, they are easily distinguishable by two distinct peaks in the melting curves obtained from the real time apparatus.

## Results

In order to test the influence of the *H-2* murine region in the *T. cruzi* tissue tropism, we utilized initially two different clonal isolates of parasite to infect C57BL/6 (*H-2^b^*) and C57BLKS/J (*H-2* ^d^) mice. Data were collected from mice after six months infection, to simulate the chronic phase of Chagas' disease. The characterization and semi-quantitative assessment of the infecting *T. cruzi* population by LSSP-PCR in different organs of mice simultaneously infected with both JG and Col1.7G2 were used to analyze infection and tropism.

**Figure 1:**
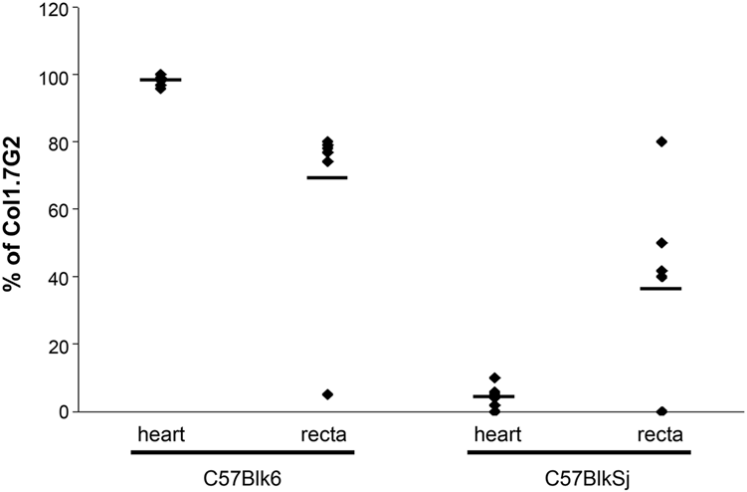
Relative percentage of the Col1.7G2 clone of *T. cruzi* in tissues of C57Blk6 (*H2^b^*) and C57BlkSj (*H2^d^*) after six months of double infection with Col1.7G2 and JG strains, using the LSSP-PCR technique. Each point indicates data from individual mice and the bar represents the median value.

The molecular characterization of the parasites in mice co-infected with JG and Col1.7G2 showed no specific differential distribution of *T. cruzi* in the recta of C57BL/6 and C57BLKS/J (Fig 1). However, a completely different result was observed for the hearts of these mice. The tissue distribution of the two *T. cruzi* populations showed a complete correlation with its genetic background. A high proportion of Col1.7G2 (99% of infecting population; n = 8) in relation to JG was always obtained in the hearts from C57BL/6, while in the hearts from C57BLKS/J mice, the proportion of Col1.7G2 was reduced to basal levels (5%; n = 8) (Fig. 1).

**Figure 2:**
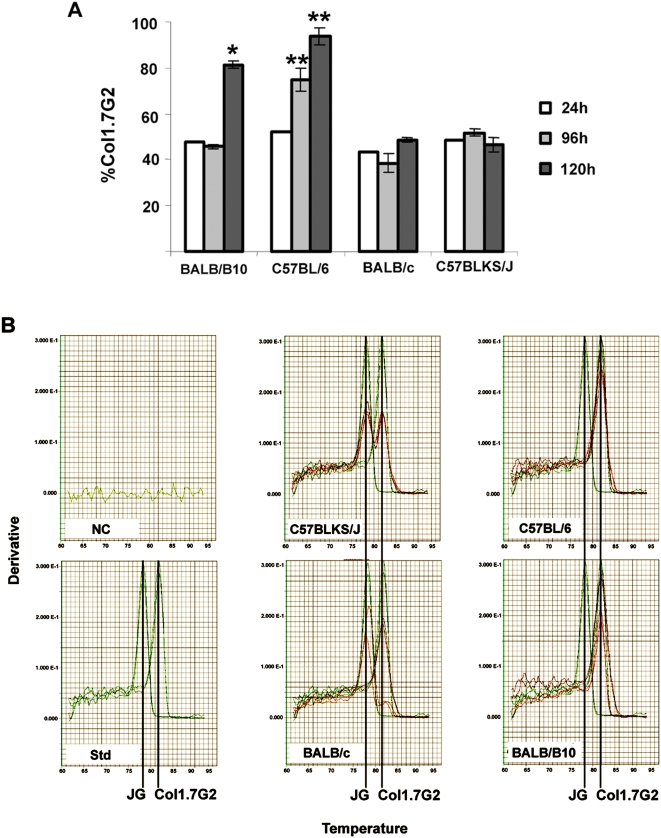
Quantitative analyses of the percentage of Col1.7G2 and/or JG after intracellular development in distinct murine cardiac explants exposed to equal mixture of trypomastigote forms of each population. (a) Relative percentage of the Col1.7G2 clone using the LSSP-PCR technique, 24, 96 and 120 h post-infection in murine cardiac explants. P values above the bars were obtained by the two-sample Student t test and indicate significant differences between explants of congenic animals. One asterisk (*) indicates statistically significant differences between BALB/B10 (*H2^b^*) and BALB/c (*H2^d^*) and two asterisks (**) between C57BL/6 (*H2^b^*) and C57BLKS/J (*H2^d^*). (b) D7 allele melting curves obtained from the real time PCR of cardiac explants of murine mice. (NC) indicates the negative control melting curves obtained from different non-infected explant samples; (Std) indicates the melting curves obtained from the real time PCR of artificial mixtures of equal amounts of JG and Col1.7G2 DNA. In all other graphs, it is shown the melting curves obtained from explants of each different mouse strains infected with a mixture of Col1.7G2 and JG. For these graphs the red curves correspond to the amount of each *T. cruzi* population (Col1.7G2 and JG) obtained from the experimentally double infected cardiac explants, while the green curves are superimposed standard curves obtained from the real time PCR of the parasite artificial mixtures. The peaks corresponding to the specific D7 alleles for each *T. cruzi* DNA population (Col1.7G2 – 81.5°C and JG – 78.2°C), are indicated with a black line.

The influence of the humoral immune response on the tissue distribution pattern of *T. cruzi* was assessed by using the same parasite populations to infect simultaneously cardiac explants from four mice lineages. These mice differed from each other by interchanging their *H-2* haplotypes in two different genetic backgrounds. The relative amount of each infecting parasite was initially assessed using the LSSP-PCR technique. Two parameters were analyzed: the rate of *T. cruzi* cell invasion, evaluated after 24 h of parasite incubation with the host cell, and parasite intracellular growth, analyzed four (96 h) and five days (120 h) post-infection. No difference in the relative amount of JG and Col1.7G2 was observed after 24 h of infection in all mouse lineages (Fig. 2a). In addition, no difference in intracellular growth of both *T. cruzi* populations was observed in heart explants from C57BLKS/J and BALB/c, which share the *H-2* ^d^ haplotype. However a clear predominance of the Col1.7G2 clone was observed in explants from mice with *H-2* ^b^ haplotype (C57BL/6 and BALB/B10) after 96 h or 120 h respectively (Fig. 2a). Similar results were also seen by using real time PCR of the D7 rDNA alleles of *T. cruzi* where a clear selection of Col1.7G2 was observed during parasite growth in *H2^b^* haplotype cardiac explants (Fig. 2b).

## Discussion

One of the most intriguing characteristics of Chagas disease is its broad range of clinical manifestations. Although the exact causes of this variability remain to be elicited, data from our group have demonstrated that, in mice, the MHC gene region could be involved with the distribution of different *T. cruzi* strains into different tissues [3]. The role of MHC haplotypes at different levels of the interaction of *T. cruzi* and hosts has been discussed by many authors and its supposed influence ranges from the humoral response against *T. cruzi* [10] and induction of antigen presentation to CD8^+^ T cells[11] through modulation of the expression of MHC class II molecules by the parasite [12]. While some authors saw none or few statistically significant influence of MHC locus on development of the different clinical forms of Chagas disease in patients from Brazil [13], others identified a correlation with either seropositivity or the development and severity of the clinical manifestations in humans, as well as the infection in animal models. Borrás et al. (2006), studying patients from Argentina showed a statistically significant correlation of HLA and the level of serologically positive individuals for *T. cruzi* [14]. Although not statistically significant they also found a trend for the development of the Chagas cardiomyopathy. Other studies with Mexican chagasic patients suggested that some MHC alleles could be associated with the clinical forms of chronic Chagas disease especially with the higher risk of heart disease development [15]. Furthermore molecular class II MHC typing among chagasic patients has allowed the identification of putative MHC susceptibility genes in *T. cruzi* seropositive individuals with cardiac disease, as compared with non symptomatic individuals [16]. In the murine model, there are also indications that the MHC region modulates tissue damage, host survival[17] and the susceptibility/resistance to *T. cruzi* infection [18]. Additionally, there is a QTL for tissue burdens of *T. cruzi* that covers the MHC *loci* and a large flanking region [19]. In the present work, we sought to investigate further the influence of MHC in the murine model of Chagas disease using *in vivo* and *in vitro* approaches.

*In vivo* experiments using congenic mice [C57BLKS/J (*H-2* ^d^) and C57BL/6 (*H-2* ^b^)] infected with the *T. cruzi* monoclonal populations (JG and Col1.7G2) demonstrated that the percentage of positive tissue samples was always higher in C57BLKS/J independent of the parasite strain (83% in C57BLKS/J versus 57% in C57BL/6). This suggests that some alleles linked to *H-2* ^d^ haplotype could lead to a higher susceptibility to Chagas disease. In addition to its apparent influence in the mice susceptibility to *T. cruzi*, the MHC haplotype seemed to be also important for the differential tissue distribution of parasite populations. Even though we were not able to specifically access the total parasite burden per sample, we observed a notable predominance of Col1.7G2 in relation to JG in the hearts of C57BL/6. This predominance, however, was totally inverted when we exchanged the MHC haplotype from *H-2* ^b^ to *H-2* ^d^ in the same mouse background (C57BLK/6 to C57BLKS/J). Similar results were obtained previously [3], where hearts from DBA-2 and BALB/c mice, both presenting the *H-2* ^d^ haplotype, were mainly infected by JG, while hearts from C57BL/6 were colonized exclusively by Col1.7G2. This apparent interference of the MHC haplotype in *T. cruzi* tissue tropism was particularly obvious in hearts. No clear difference in the parasite distribution was detected in the recta of the same animals, as in both mice lineages, we could find recta infected with either only the JG strain or the Col1.7G2 clone, or a mixture of both parasite populations. Therefore the importance of the MHC gene region in determining parasite tissue tropism seems to be dependent on the infected organ.

Data obtained from cardiac explants also support the *in vivo* results. Analyses of the relative amount of parasites during the mixed infection over a course of 5 days in cardiac explants showed an increase of Col1.7G2 in relation to JG in the cultures from C57BL/6 and BALB/B10 mice, both presenting *H-2* ^b^ haplotype, but did not in animals with the *H-2* ^d^. This predominance was not observable in the first 24 h, but could only be noticed later during the infection suggesting that this difference was the result of better or faster multiplication of Col1.7G2 in hearts from *H-2* ^b^ mice rather than a higher invasion rate. In accordance with these observations, we propose the *H-2* or related factors that result in the differential colonization have an intracellular nature. *H-2^d^* doubly infected cardiac explants (obtained from C57BLKS/J and BALB/c), on the other hand, did not show a difference in intracellular growth of both *T. cruzi* populations, as observed previously in *in vivo* experiments [3], at least not for the time course analyzed (96 hs). In fact parasite strain selection in BALB/c and DBA-2 mice infected with both JG and Col1.7G2, was only observed three to six months post infection (experimental chronic phase). It is likely that there is a difference in the kinetics of parasite growth depending on the strain and MHC background. Our data do not allow us to determine the intracellular mechanisms of this parasite selection phenomenon, however susceptibility to other intracellular protozoan parasites, such as *Toxoplasma gondii* and *Leishmania amazonensis* is also affected by the *H-2* haplotypes [20], [21]. Furthermore, it remains to be established whether differences in tissue tropism of different parasite strains are due to the MHC haplotype directly or to any other gene selected through linkage disequilibrium with this locus. Some of these genes are not related to the immune system or even have not been identified [22].

Concluding, our observations demonstrate a strong influence of the *H-2* region on the differential tissue tropism of *T. cruzi* populations in mice, confirming our previous hypothesis [3]. Even though the mouse model do not reproduce actual clinical manifestation of the disease, in our previous studies, selection did influenced the outcome of inflammation and tissue damage [2], [3]. Also previous studies with chronic digestive chagasic patients showed that parasites were only detected in oesophageal samples from patients with megaesophagus and not from the ones with megacolon [22]. Thus in accordance with the histotropic-clonal model of Chagas disease [23], the differential tissue tropism of the parasites, by its turn, could conceivably determine the variety of clinical manifestation exhibited by chagasic patients.
